# Identifying biomarkers of papillary renal cell carcinoma associated with pathological stage by weighted gene co-expression network analysis

**DOI:** 10.18632/oncotarget.15842

**Published:** 2017-03-02

**Authors:** Zhongshi He, Min Sun, Yuan Ke, Rongjie Lin, Youde Xiao, Shuliang Zhou, Hong Zhao, Yan Wang, Fuxiang Zhou, Yunfeng Zhou

**Affiliations:** ^1^ Hubei Cancer Clinical Study Center, Hubei Key Laboratory of Tumor Biological Behaviors, Wuhan, China; ^2^ Department of Radiation and Medical Oncology, Zhongnan Hospital of Wuhan University, Wuhan, China; ^3^ Hubei Cancer Clinical Study Center, Zhongnan Hospital of Wuhan University, Wuhan, China; ^4^ Department of Oncology, Zhongnan Hospital of Wuhan University, Wuhan, China

**Keywords:** papillary renal cell carcinoma (PRCC), the cancer genome atlas (TCGA), weighted gene co-expression network analysis (WGCNA), survival prognosis, pathological stage

## Abstract

Although papillary renal cell carcinoma (PRCC) accounts for 10%–15% of renal cell carcinoma (RCC), no predictive molecular biomarker is currently applicable to guiding disease stage of PRCC patients. The mRNASeq data of PRCC and adjacent normal tissue in The Cancer Genome Atlas was analyzed to identify 1148 differentially expressed genes, on which weighted gene co-expression network analysis was performed. Then 11 co-expressed gene modules were identified. The highest association was found between blue module and pathological stage (r = 0.45) by Pearson's correlation analysis. Functional enrichment analysis revealed that biological processes of blue module focused on nuclear division, cell cycle phase, and spindle (all *P* < 1e-10). All 40 hub genes in blue module can distinguish localized (pathological stage I, II) from non-localized (pathological stage III, IV) PRCC (*P* < 0.01). A good molecular biomarker for pathological stage of RCC must be a prognostic gene in clinical practice. Survival analysis was performed to reversely validate if hub genes were associated with pathological stage. Survival analysis unveiled that all hub genes were associated with patient prognosis (*P* < 0.01). The validation cohort GSE2748 verified that 30 hub genes can differentiate localized from non-localized PRCC (*P* < 0.01), and 18 hub genes are prognosis-associated (*P* < 0.01).

ROC curve indicated that the 17 hub genes exhibited excellent diagnostic efficiency for localized and non-localized PRCC (AUC > 0.7). These hub genes may serve as a biomarker and help to distinguish different pathological stages for PRCC patients.

## INTRODUCTION

Kidney malignant tumor is a heterogeneous disease of which epithelial renal cell carcinoma (RCC) constitutes the vast majority [1]. Based on morphological features, RCC can be divided into multiple histological subtypes, encompassing clear cell, papillary, chromophobe, collecting duct, and unclassified subtypes [2]. Up to one-third of patients with RCC already suffer with a distant metastasis at the time of diagnosis [3]. Papillary RCC (PRCC), taking up about 10%–15% of RCC, is the second most common subtype. At present, no effective therapeutic approach is available for patients with advanced PRCC [4]. Many biomarkers for renal clear cell carcinoma have been discovered, including *VHL, VEGF, CAIX* and *HIF1a/2*a mutations, some of which could predict therapeutic effect and clinical prognosis [5]. However, PRCC's molecular biomarkers for predicting curative effect and prognosis have rarely been reported [6]. Thus, it is necessary to identify novel molecular biomarkers that can predict disease stage and clinical outcome of PRCC patients, which could help understand its pathogenesis and provide personalized treatment.

Rapid technological breakthroughs of genome-wide sequencing have shed new light on the research of clinical issues and related pathological mechanisms in various cancers [7]. The Cancer Genome Atlas (TCGA), a large integrated collection of clinical information and gene sequencing data, allow for systematic analysis for underlying molecular mechanisms of various clinical features associated with cancers, e.g. pathological stage, histological type, tumor grade, diagnosis and prognosis, contributing to improvements in diagnostic methods and ultimately ameliorating the survival prognosis of cancer patients [8]. Weighted gene co-expression network analysis (WGCNA) can construct free-scale gene co-expression networks to explore the relationships between different gene sets or between gene sets and clinical features [9]. WGCNA has been widely applied to finding the hub genes associated with clinical feature in different cancer types. For example, *PS15A*, *PTGDS*, *CD53* and *MSI2* have been identified as potential therapeutic targets or diagnostic biomarkers for uveal melanoma [10]. *COL5A2*, *HOXB1*, *CENP-E*, *MYCN* and *BCL-2* were predicted to be associated with endometrial cancer progression via Hedgehog signaling and other cancer-related pathways [11]. Additionally, *SRASSF2* and *CDCA7* were identified as potential biomarkers for retinoblastoma [12].

In this study, WGCNA and other analysis methods are adopted to jointly analyze clinical information and mRNASeq data of PRCC patient samples provided by TCGA data set to identify key genes associated with clinical features. These key genes may have important clinical implications and serve as diagnostic and prognostic biomarkers or therapeutic targets.

## RESULTS

### Preparation of clinical and genetic data

**Figure 1:**
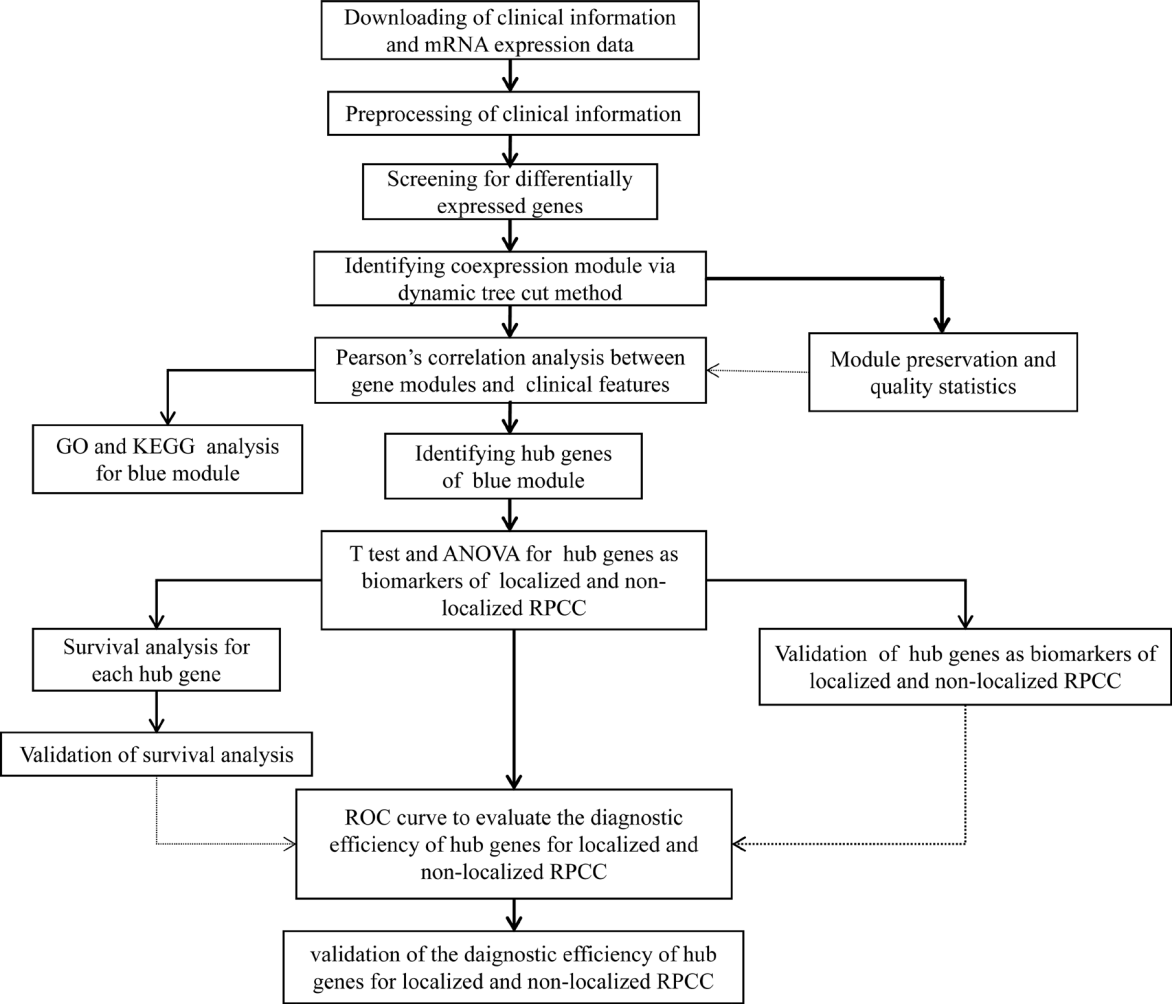
Flow chart of data preparation, processing, analysis and validation in this study

A workflow of this study is shown in Figure 1. In the TCGA data set, mRNA sequencing data contained 32 normal renal samples and 290 PRCC samples, level-4 clinical data comprised 291 PRCC patients samples. Standardized level-3 RNAseq data was utilized for prognostic analysis. After eliminating cases without complete follow-up information, 289 patients remained available for prognostic analysis. Raw level-3 RNAseq data was utilized for differential expression analysis and WGCNA. After excluding patients without complete clinical information or explicit T stage, 106 patients were included in the WGCNA analysis. In computer language, clinical data, originally described as character, was encoded to numeric form for WGCNA analysis. Original and numeric clinical information, as well as summarized data of the PRCC patients in TCGA were displayed in Supplementary Table 1. In the validation cohort GSE2748, there were 34 patients with pathological stage information and 29 patients with prognostic data. Clinical features of the PRCC patients in GSE2748 were shown in Supplementary Table 2.

### Screening for differentially expressed genes (DEGs)

**Figure 2:**
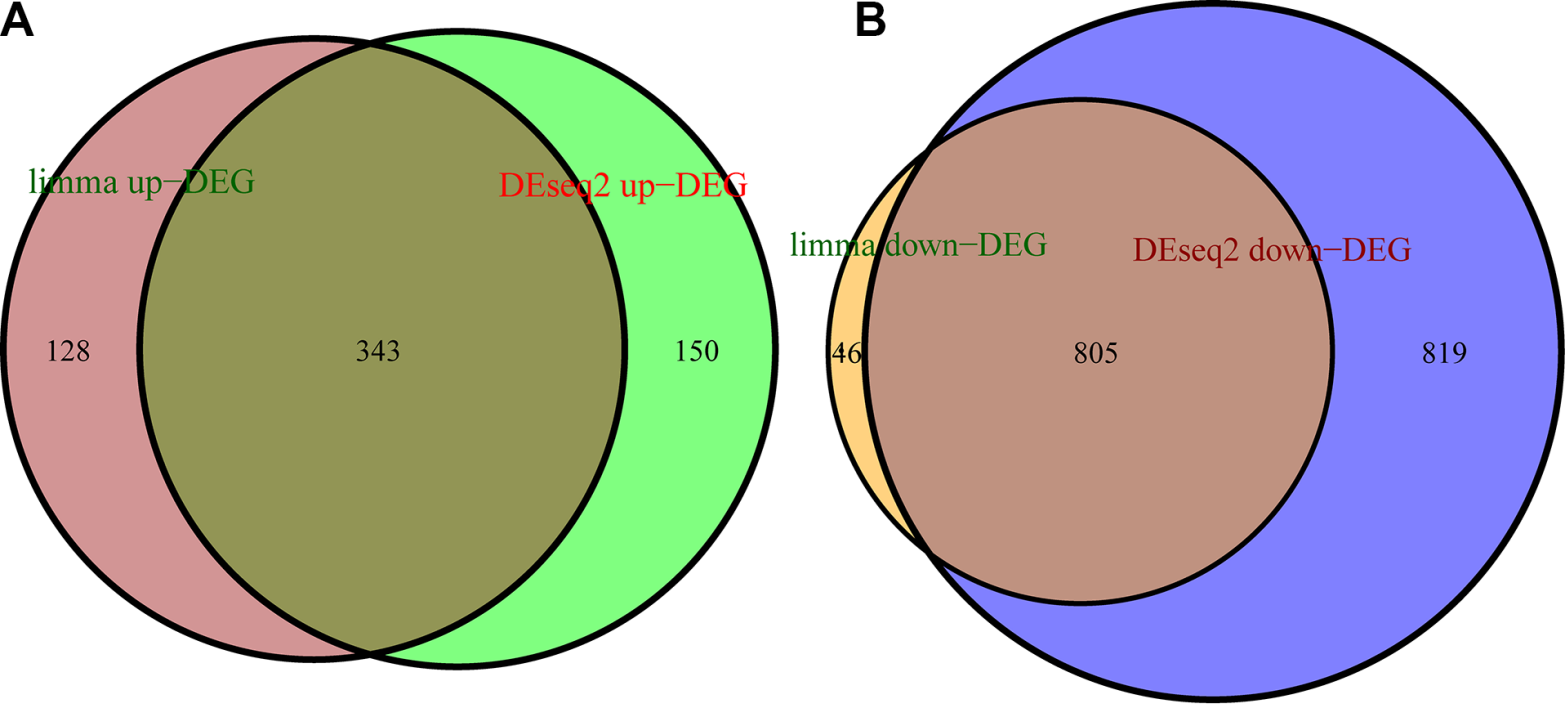
DEGs were screened with limma and DESeq2 algorithms (**A**) number of up-regulated DEGs identified with limma (brown circle) and DESeq2 (green circle), and overlapping DEGs (auburn). (**B**) number of down-regulated DEGs identified with limma (orange circle) and DESeq2 (blue circle), and overlapping DEGs (light-brown).

Raw level-3 RNAseq data of 19,405 mRNAs of 290 PRCC tissue and 32 adjacent non-tumor tissue samples was subjected to DEG analysis. DEGs were screened by DESeq2 [13] and limma [14] algorithms. 2117 DEGs were identified by DESeq2, among which 493 were up-regulated in cancer samples and 1624 down-regulated. 1322 DEGs were identified by limma, among which 471 were up-regulated in cancer samples and 851 down-regulated. Then a total of 1148 overlapping DEGs were obtained by both algorithms, among which 343 were up-regulated and 805 down-regulated, accounting for 29.94% and 70.06% of the total overlapping differential genes, respectively (Figure 2).

### Co-expression network construction and module preservation analysis

**Figure 3:**
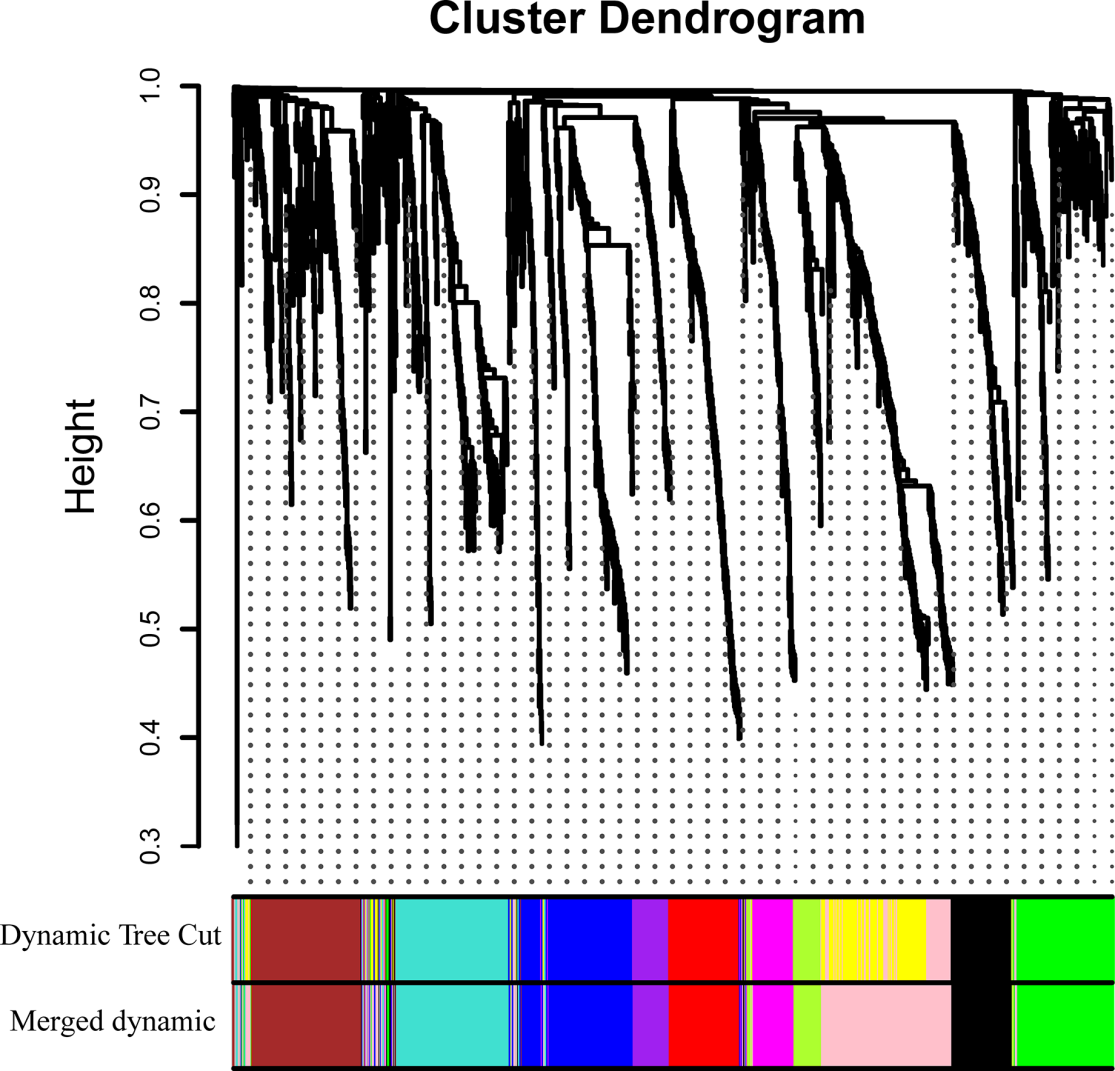
Clustering dendrograms of genes Gene clustering tree (dendrogram) obtained by hierarchical clustering of adjacency-based dissimilarity. The colored row below the dendrogram indicates module membership identified by the dynamic tree cut method, together with assigned merged module colors and the original module colors.

WGCNA was performed on 1148 DEGs of 106 samples. After discarding four outlier samples, the connectivity between genes in the gene network met a scale-free network distribution when the soft threshold power beta was set to 4 (Supplementary Figure 1). Then 11 co-expressed modules, ranged in size from 46 to 206 genes (assigning each module a color for reference), were identified. While the “grey” module was reserved for genes identified as not co-expressed (Figure 3). The genes in each module is listed in Supplementary Table 3.

**Figure 4:**
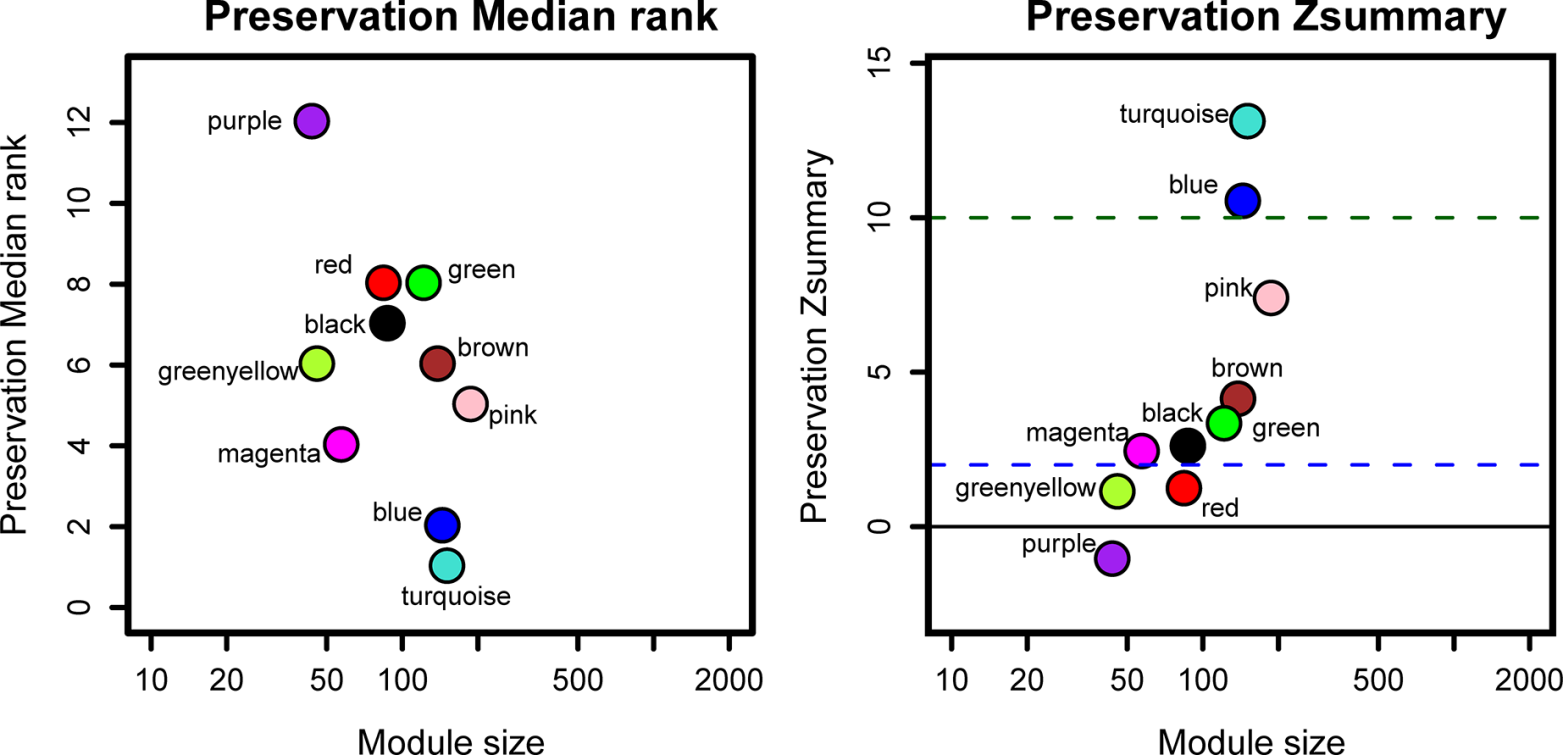
The medianRank and Zsummary statistics of the module preservation of the DEG modules In the preservation medianRank graph on the left, the medianRank of the modules close to zero indicates a high degree of module preservation. In the preservation Zsummary graph on the right, the dashed blue and green lines indicate the thresholds Z = 2 and Z = 10, respectively. These horizontal lines indicate the Zsummary thresholds for strong evidence of conservation (above 10) and for low to moderate evidence of conservation (above 2).

By comparing the TCGA data set with the test data set GSE2748, the summary preservation statistics [15], a statistics that determined whether a reference network can be found in another test network, were visualized. blue and turquoise modules were found to be most stable. Whereas the rest modules were not stable enough with their Zsummary statistics below 10. The median Rank statistics for blue and turquoise modules presented the minimum, suggesting that their preservation tended to be best among all modules (Figure 4).

### Finding module of interest and functional annotation

**Figure 5:**
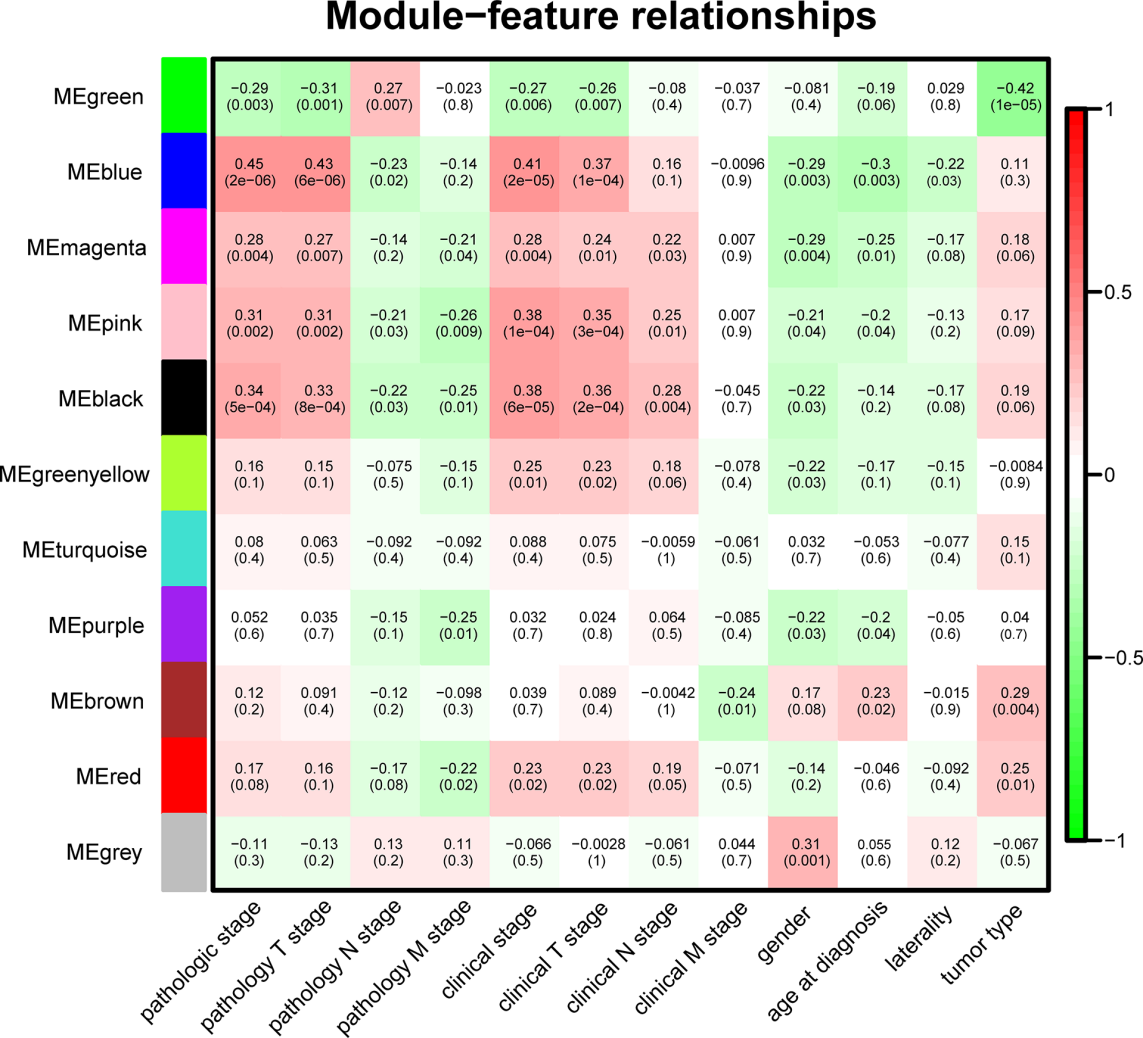
Module-feature associations Each row corresponds to a module Eigengene and each column to a clinical feature. Each cell contains the corresponding correlation in the first line and the *P*-value in the second line. The table is color-coded by correlation according to the color legend.

It is of great biological significance to identify modules most significantly associated with clinical features. The highest association in the Module-feature relationship was found between blue module and pathological stage (r = 0.45, *P* = 2 × 10^−6^; Figure 5), which were selected as module of interest and clinical feature to be studied in subsequent analyses. The module of interest was also associated with pathology T stage, clinical stage and clinical T stage. The second-highest association in the Module-Trait relationship was found between green module and tumor type (r = 0.42, *P* = 1 × 10^−5^), but the module was not further analyzed because the preservation statistics indicated that it was not stable enough.

**Figure 6:**
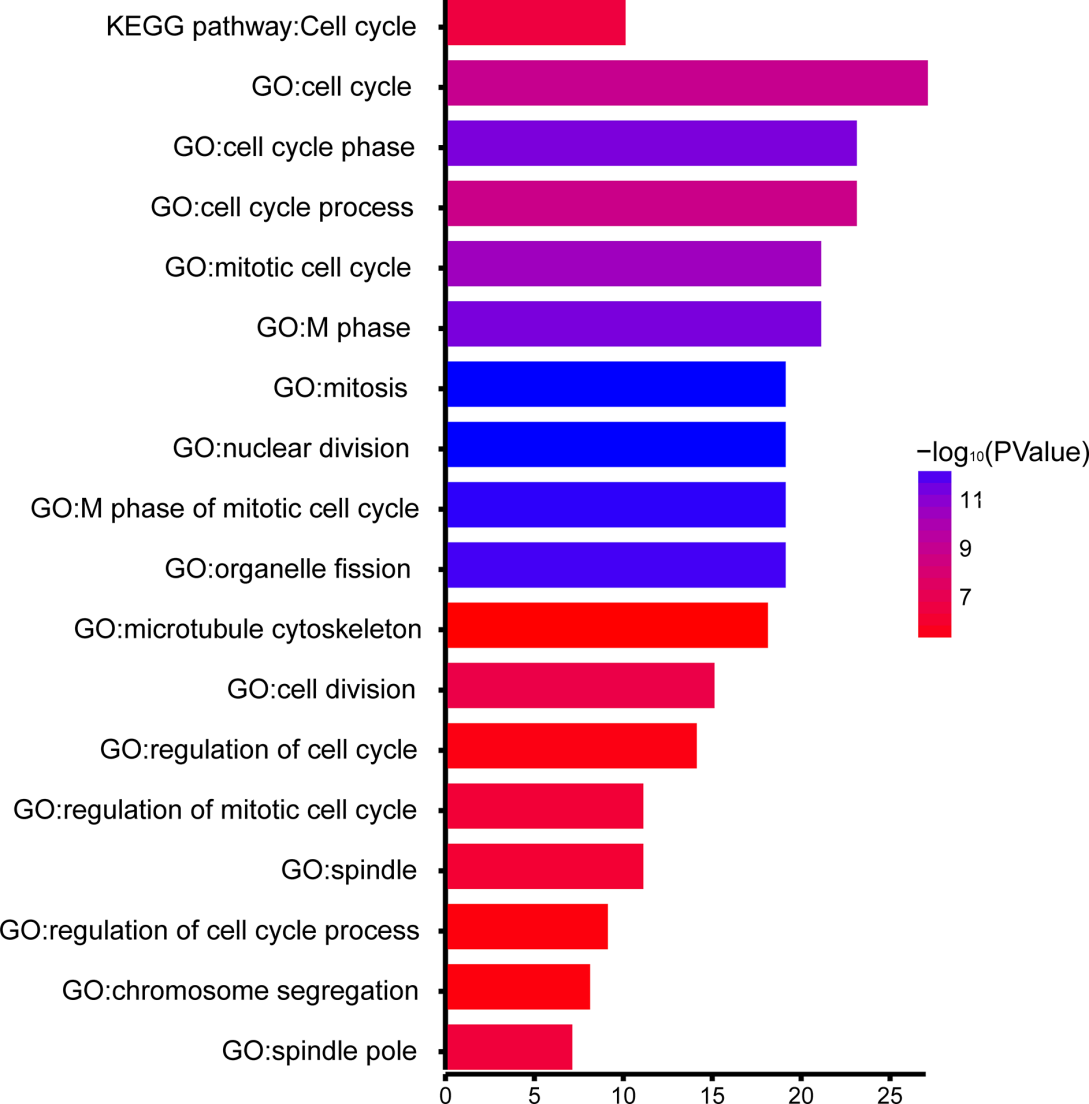
GO functional and KEGG pathway enrichment analyses for genes in the object module The x-axis shows the number of genes and the y-axis shows the GO and KEGG pathway terms. The -log10 (*P*-value) of each term is colored according to the legend.

In order to explore biological relevance of blue module, 154 genes in blue module were mapped into the DAVID database [16] and subjected to Gene Ontology (GO) functional and KEGG pathway enrichment analyses. Biological processes of blue module were found to focus on nuclear division (*P* = 3.72×10^−13^), cell cycle phase (*P* = 4.59 × 10^−12^), mitosis (*P* = 3.72 × 10^−13^), and the spindle (*P* = 7.92 × 10^−11^). However, in KEGG pathway analysis, cell cycle was identified as only significant pathway. (*P* = 3.53 × 10^−7^; Figure 6).

### Identifying hub genes and correlation analysis

Forty hub genes, which exhibited high intramodular connectivity in the module of interest and high gene significance for pathological stage, were identified in the blue module.

Significant difference (*P* < 0.01) of each hub gene was found across different pathological stages with one-way ANOVA. When an independent *t*-test was utilized, difference for each hub gene between pathological stage I and II, or between stage III and IV was not always significant, but difference for each hub gene between localized (pathological stage I, II) and non-localized PRCC (pathological stage III, IV) was found to be persistently statistically significant (*P* < 0.01). ANOVA and *t*-test analysis on hub genes between different pathology T stages got similar results, where significant differences were found between pathology T1/T2 and T3/T4 group.

The relationship between all hub genes and pathological stage was shown in Supplementary Figure 2, and the relationship between all hub genes and pathology T stage in Supplementary Figure 3. The correlation between hub genes and pathological stage was verified by the validation set GSE2748. Significant differences were found for 30 hub genes between localized and non-localized PRCC, shown in Supplementary Figure 4.

### Survival analysis on hub genes

The mRNASeq data and follow-up information of 289 PRCC patients in TCGA were subjected to survival analysis [17]. We found that all hub genes were associated with patient prognosis (*P* < 0.01). When the results of survival analysis was verified by the validation set GSE2748, 18 hub genes were still prognosis-associated (*P* < 0.05). Positive results of the survival analysis were shown in Supplementary Figure 5 for 40 hub genes in the blue module and in Supplementary Figure 6 for 18 hub genes in GSE2748.

### Efficacy evaluation for hub genes

ROC curve analysis was implemented to evaluate the diagnostic efficiency of hub genes to distinguish between localized and non-localized PRCC [18]. AUC values for 40 hub genes were greater than 0.7 in TCGA data set. In the validation set GSE2748, only 17 hub genes are prognosis-associated and capable of discriminating localized and non-localized PRCC. We calculated the AUC value and plotted ROC curve for the 17 hub genes. Each AUV value of the 17 hub genes was bigger than 0.7. The ROC curves for 40 hub genes were shown in Supplementary Figure 7 and 17 hub genes shown in Supplementary Figure 8.

## DISCUSSION

In the present study, we identified 17 candidate biomarkers for PRCC by applying WGCNA, a systems biology method, and other analysis methods on mRNASeq data and clinical information of PRCC patients in TCGA for the first time. We found that the 17 biomarkers can distinguish between localized (pathological stage I, II) and non-localized PRCC (pathological stage III, IV), which was verified by a microarray-based validation cohort GSE2748. The findings may contribute to the improvement of therapeutic decision-making, risk stratification and prognosis prediction for PRCC patients.

TCGA provides both clinical information and gene sequencing data from a large number of patient samples in many cancer types. Genetic data, coupled with clinical information, is an advantage when compared with other databases such as Oncomine, SEER, or GEO data sets. Numerous studies utilized the data of RCC in TCGA. But most of them focused on renal clear cell carcinoma, only four on PRCC. Two publications concentrating on the histological subtype of PRCC revealed that type I and type II PRCC exhibited difference in clinical and biological characteristics, and that type II PRCC consisted of at least three subtypes based on molecular and phenotypic features [19, 20]; One study found three specific miRNAs associated with the progression and aggressiveness of PRCC [21]; The fourth study proposed an immunoscoring approach based on RNASeq data of PRCC [22]. The four studies didn't fully exploit clinical information of PRCC patients in TCGA data set, or didn't adopted coexpression network analysis to widely screen biomarkers associated with clinical features.

WGCNA provides a global interpretation of gene expression information by constructing gene co-expression networks on the basis of similarities of expression profiles among samples. Many articles related to WGCNA have been published on prestigious journals in the field of biological information and systems biology [23–25]. WGCNA algorithm has been applied to identifying related gene, biological pathway and tumor therapeutic target for complex diseases, such as familial combined hyperlipidemia [26], Alzheimer's disease [27], and osteoporosis [28]. Considerable amounts of tumor and control samples with genetic data and corresponding clinical information in TCGA offer promising opportunities to employ WGCNA for cancer research. However, few studies have mined the TCGA database with this method. For example, data on four different cancers, ovarian, breast, lung and skin, was processed with WGCNA to compare patterns of co-expressed genes in tumors grouped according to their *TP53* missense or null mutation status. Examining mutation-type-related changes in correlated sets of genes might provide new insights into tumor biology [29]. To our knowledge, mining PRCC data in TCGA to explore the correlation between gene expression profiles and clinical features has not been previously reported.

The purpose of our study was to mine mRNASeq data and clinical information of PRCC patients in TCGA with WGCNA to find out biomarkers associated with clinical features. In cancer research, candidate biomarkers should correctly distinguish cancerous from normal tissues. Differentially expressed genes were obtained when comparing PRCC samples with normal renal tissue samples, on which WGCNA was performed. Then 11 co-expression modules were identified via the dynamic tree cut method. By means of correlating gene modules with clinical features, highest positive correlation was found between blue module and pathological stage. The summary preservation statistics approved that blue module was one of the most stable modules. After the previous analysis, blue module was considered as a gene set with clinical significance. A range of genes with the highest connectivity in module was defined as hub genes that largely determined characteristics of the module. Exploring the relationship between blue module and pathological stage could be simplified as to find out the connection between hub genes in blue module and pathological stage, so as to seek genes with important biological significance. Forty hub genes were screened out in blue module.

Enrichment analyses for blue module indicated that biological processes of blue module focused on nuclear division, cell cycle phase, mitosis, spindle, etc. Previous studies have unveiled that hub genes of the blue module played vital role in the formation of other cancers. BUB1 has been reported to exert a direct effect on the suppression of p53-mediated cell death via physical interaction with p53 at kinetochores in response to mitotic spindle damage [30]. Overexpression of BUB1 was linked with poor outcomes in breast cancer patients [31]. Microtubule-associated protein TPX 2, which could bind to tubulin and induce microtubule polymerization, was crucial for mitotic spindle formation [32]. Aberrant expression of *TPX2* may be essential in both malignant transformation of respiratory epithelium and progression of squamous cell lung cancer [33]. No reports concerning the relationship between these hub genes and pathological stage of PRCC have been published. However, some of them exhibited a close relationship with disease stage of other cancers in previous studies. For example, *BUB1* mRNA was significantly co-expressed with *AURKB* mRNA in advanced-stage ovarian serous carcinoma [34]. Another study found that the circulating *CCNB2* mRNA level in serum was significantly correlated with cancer stage and metastasis status [35].

By means of one-way ANOVA and an independent sample ***t*** test, the mRNASeq expression of all hub genes can effectively distinguish localized PRCC (pathological stage I or II) from non-localized PRCC (pathological stage III or IV), also can successfully recognize the pT1/pT2 group from the pT3/pT4 group. Hub genes distinguish different pathological stages in PRCC possibly because the pathology T stage greatly affects the pathological stage. The data in the validation set GSE2748 confirmed that 30 hub genes could make a distinction between localized and non-localized PRCC. These hub genes might be good biomarkers for distinguishing between localized and non-localized PRCC.

The pathological stage of RCC is the most effective prognostic factor [36]. The five-year survival rate of non-localized RCC is significantly lower than that of localized RCC [36]. Patients with higher pathological stages tend to have worse prognosis. Theoretically, genes related to pathological stage are supposed to be associated with prognosis. Conversely, if these genes are not related to prognosis, they should not belong to the genes associated with pathological stage. Survival analysis was performed to reversely validate if hub genes were associated with pathological stage. Survival analysis demonstrated that all 40 hub genes were significantly associated with prognosis (*P* < 0.01). But only 18 hub genes were prognosis-related genes in the validation cohort GSE2748. By seeking overlapped genes, 17 hub genes were correlated with pathological stage and prognosis at the same time, no matter in the TCGA data set or in the validation set GSE2748. Additionally, ROC curve indicated that the 17 hub genes exhibited excellent diagnostic efficiency for localized and non-localized PRCC (AUC > 0.7). To the best of our knowledge, this is the first time to identify 17 hub genes as biomarkers capable of distinguishing localized from non-localized PRCC.

Some limitations of this study should be mentioned. The most vital genes out of 17 hub genes can't been filtered out due to the restrictions of the bioinformatics methods. A large number of clinical samples are required to validate our findings and elucidate the underlying mechanisms of how these hub genes impact on pathological stage.

In summary, WGCNA and other method are adopted to analyze RNAseq data and clinical information of PRCC patient in TCGA, a set of 17 biomarkers capable of distinguishing localized from non-localized PRCC are identified. These results are of great clinical significance and will contribute to personalized therapy.

## MATERIALS AND METHODS

### Collection of clinical and genetic data

RNA sequencing data sets and clinical information of kidney PRCC patients were downloaded from the TCGA repository website (http://firebrowse.org/). Level-3 RNAseq data was derived from Illumina HiSeq RNAseq v2 RSEM genes. Microarray-based normalized mRNA data sets of PRCC patients in GSE2748, which served as a independent validation cohort, were obtained from the Gene Expression Omnibus. Clinical information of PRCC patients in GSE2748 were extracted from a published literature [38]. Microarray expression data of GSE2748 was annotated according to the Affymetrix Human Genome U133 Plus 2.0 Array platform. Data processing in this study met the human subject protection and data access policies set by NIH and TCGA, respectively. Clinical follow-up data of PRCC patients in TCGA were retrieved for prognostic analysis. Other clinical information, including AJCC pathological TNM stage (pathological stage, pT, pN and pM), AJCC clinical TNM stage (clinical stage, cT, cN and cM), gender, age at initial pathological diagnosis and tumor type (type I or II), was extracted for WGCNA analysis.

### Screening for differentially expressed genes

Two R packages, DEseq2, based on a negative binomial distribution method [13], and limma, based on linear models and empirical Bayes methods [14], were utilized to screen DEGs between normal and cancer samples. The DEG threshold was set at a log2FoldChange > 2 and an adj.P.Val < 0.05. In order to ensure that normal and cancer samples could be well characterized by acquired DEGs, overlapping genes with significant differences obtained from both algorithms were selected as target genes to be further analyzed. .

### Gene co-expression network construction and module preservation analysis

Scale-free gene co-expression networks were constructed by the WGCNA package [9]. To ensure that the results of network construction were reliable, outlier samples were removed. An appropriate soft threshold power was selected in accordance with standard scale-free networks, with which adjacencies between all differential genes were calculated by a power function. Then, the adjacency was transformed into a topological overlap matrix (TOM), and the corresponding dissimilarity (1-TOM) was calculated. Module identification was accomplished with the dynamic tree cut method by hierarchically clustering genes using 1-TOM as the distance measure with a deepSplit value of 2 and a minimum size cutoff of 30 for the resulting dendrogram. Highly similar modules were identified by clustering and then merged together with a height cut-off of 0.25. To test the stablity of each identified module, module preservation and quality statistics were computed with the modulePreservation function (nPermutations = 200) implemented in the WGCNA package [15]. The test dataset contained microarray-based mRNA expression of 34 samples in GSE2748 [38].

### Finding module of interest and functional annotation

The correlation between modules and clinical features was evaluated by pearson's correlation tests to search biologically meaningful modules. The module and clinical feature, which exhibited the highest correlation, were selected as module of interest and clinical feature to be studied. In order to explore the potential mechanism of how module genes impact correlative clinical feature, all genes of module of interest were mapped into the DAVID database and subjected to GO functional and KEGG pathway enrichment analysis [16]. A *P*-value < 0.01 and false discovery rate (FDR) < 0.01 were set as the cutoff criteria.

### Identifying hub genes and correlation analysis

Genes with high gene significance (GS) and high module membership (MM) were defined as hub genes. Based on GS and MM, the function “networkScreening” in the WGCNA package was applied to screen hub genes in module of interest. Preliminary relationships between hub genes and corresponding clinical features were shown by boxplot graphs. Correlation between them was tested with one-way ANOVA and an independent sample *t*-test. These results were verified by the validation cohort GSE2748.

### Survival analysis and efficacy evaluation

Survival analysis was performed for all hub genes. Patients were dichotomized into two groups according to the expression of each hub gene (high vs. low). R package “survival” was adopted to implement log-rank tests and plot Kaplan-Meier survival curves [17]. In order to verify if these hub genes were indeed prognosis-related genes, the validation set GSE2748 was also utilized for survival analysis. If the log-rank test for each hub gene in TCGA data set and GSE2748 showed significant statistical difference at the same time, it was considered as prognosis-associated gene. ROC curve was plotted and AUC was calculated with“ROCR” package [18]. When AUC value was greater than 0.7, the hub gene was considered capable of distinguishing localized and non-localized PRCC with excellent specificity and sensitivity. The result was confirmed by the validation set GSE2748.

## SUPPLEMENTARY MATERIALS FIGURES AND TABLES





## Abbreviations

PRCC: papillary renal cell carcinoma
RCC: Renal cell carcinoma
DEGs: differentially expressed genes
WGCNA: weighted gene co-expression network analysis
ANOVA: analysis of variance
TCGA: The Cancer Genome Atlas
GO: Gene Ontology
KEGG: Kyoto Encyclopedia of Genes AND Genomes
DAVID: the Database for Annotation, Visualization and Integrated Discovery
ROC: receiver operating characteristic
AUC: area under the ROC curve
pT: pathology T stage
pN: pathology N stage
pM: pathology M stage
cT: clinical T stage
cN: clinical N stage
cM: clinical M stage
TOM: Topological Overlap Matrix
GS: gene significance
MM: module membership
FDR: false discovery rate.
