# Supplementary material for: Identifying biomarkers of papillary renal cell carcinoma associated with pathological stage by weighted gene co-expression network analysis
Source: Oncotarget. 2017 Mar 2;8(17):27904–14. doi: 10.18632/oncotarget.15842 (PMC5438617; doi:10.18632/oncotarget.15842)
Supplement: Supplementary file 1 [file oncotarget-08-27904-s001.pdf]

Identifying biomarkers of papillary renal cell carcinoma associated with pathological stage by weighted gene co-expression network analysis

Supplementary Materials

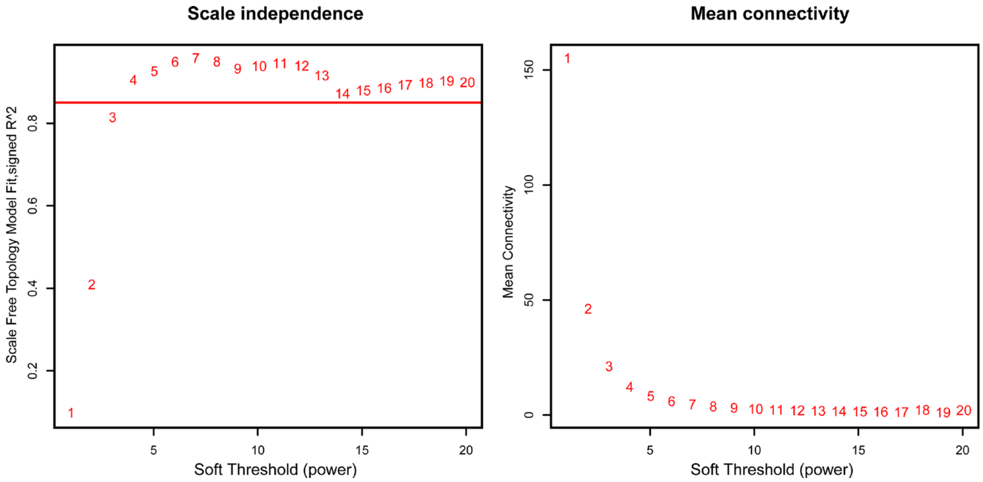

Supplementary Figure 1: Analysis of network topology for various soft-thresholding powers. The left panel shows the scale-free fit index (y-axis) as a function of the soft-thresholding power (x-axis). The right panel displays the mean connectivity (degree, y-axis) as a function of the soft-thresholding power (x-axis).

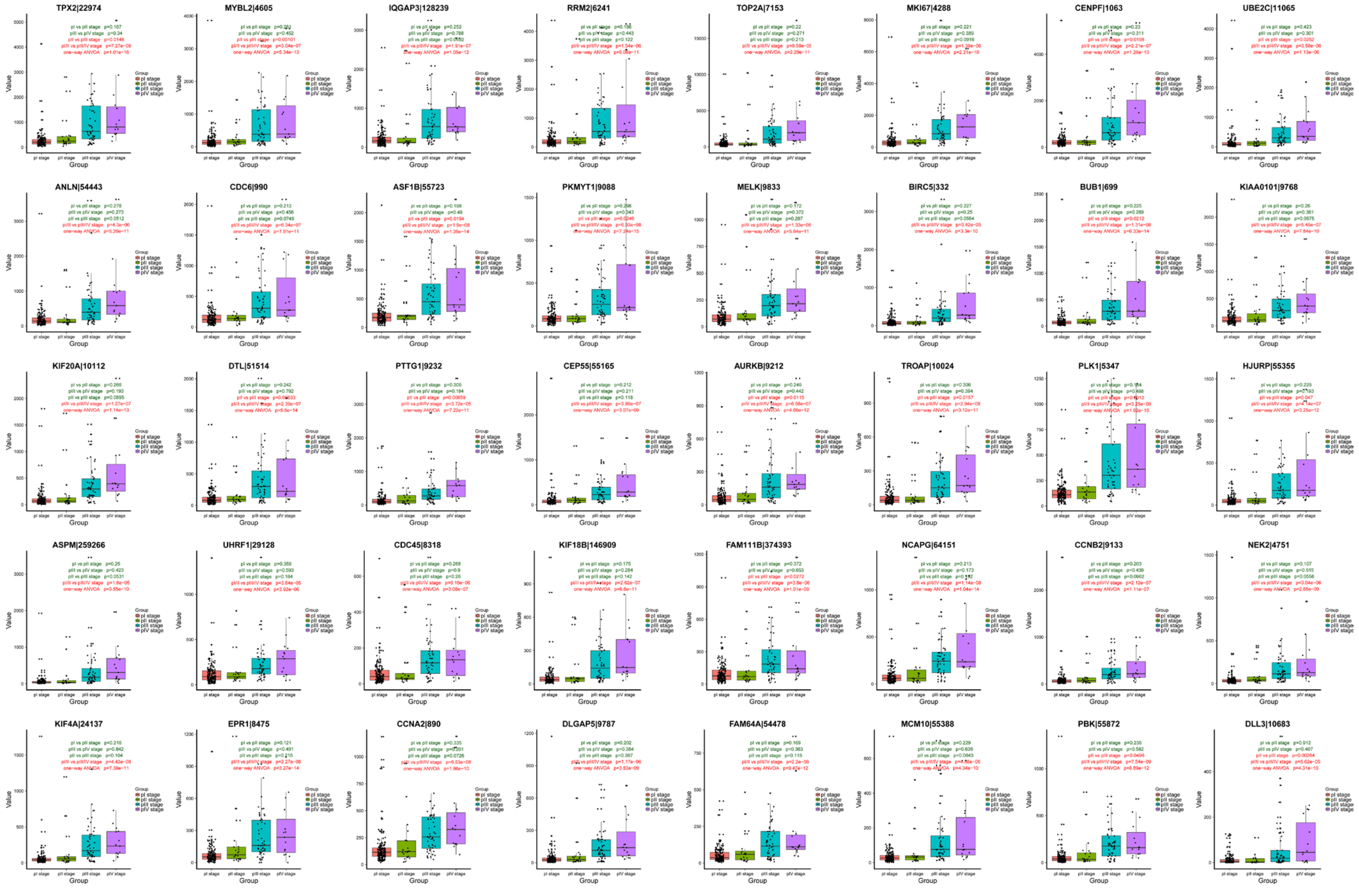

Supplementary Figure 2: Boxplots of hub genes across different pathological stages in the TCGA data set. The boxplots show the medians and dispersions of the samples of different pathological stages for each hub gene, where the scattered black spots represent the expression level of the genes. *P*-values are the results of independent sample *T*-test between pathological stage I and II, pathological stage III and IV, pathological stage II and III, pathological stage I/II and III/IV and one-way ANOVA for different pathological stages.

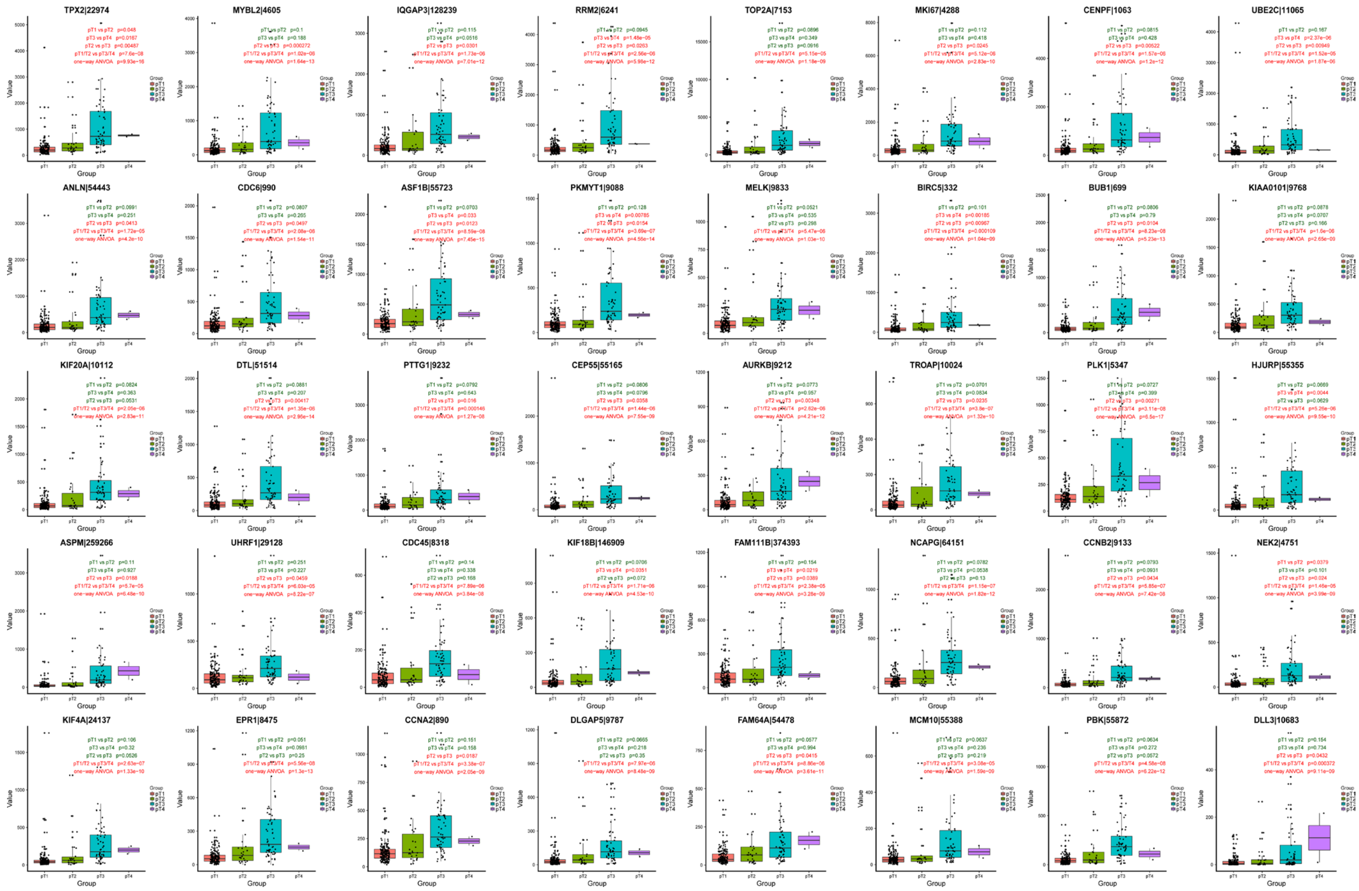

Supplementary Figure 3: Boxplots of hub genes across different pathology T stages in the TCGA data set. The boxplot shows the medians and dispersions of the samples of different pathology T stages for each hub gene, where the scattered black spots represent the expression level of the genes. *P*-values are the results of independent sample *T*-test between pT1 and pT2, pT3 and pT4, pT2 and pT3, pathology T1/T2 and T3/T4 and one-way ANOVA for different pathology T stages.

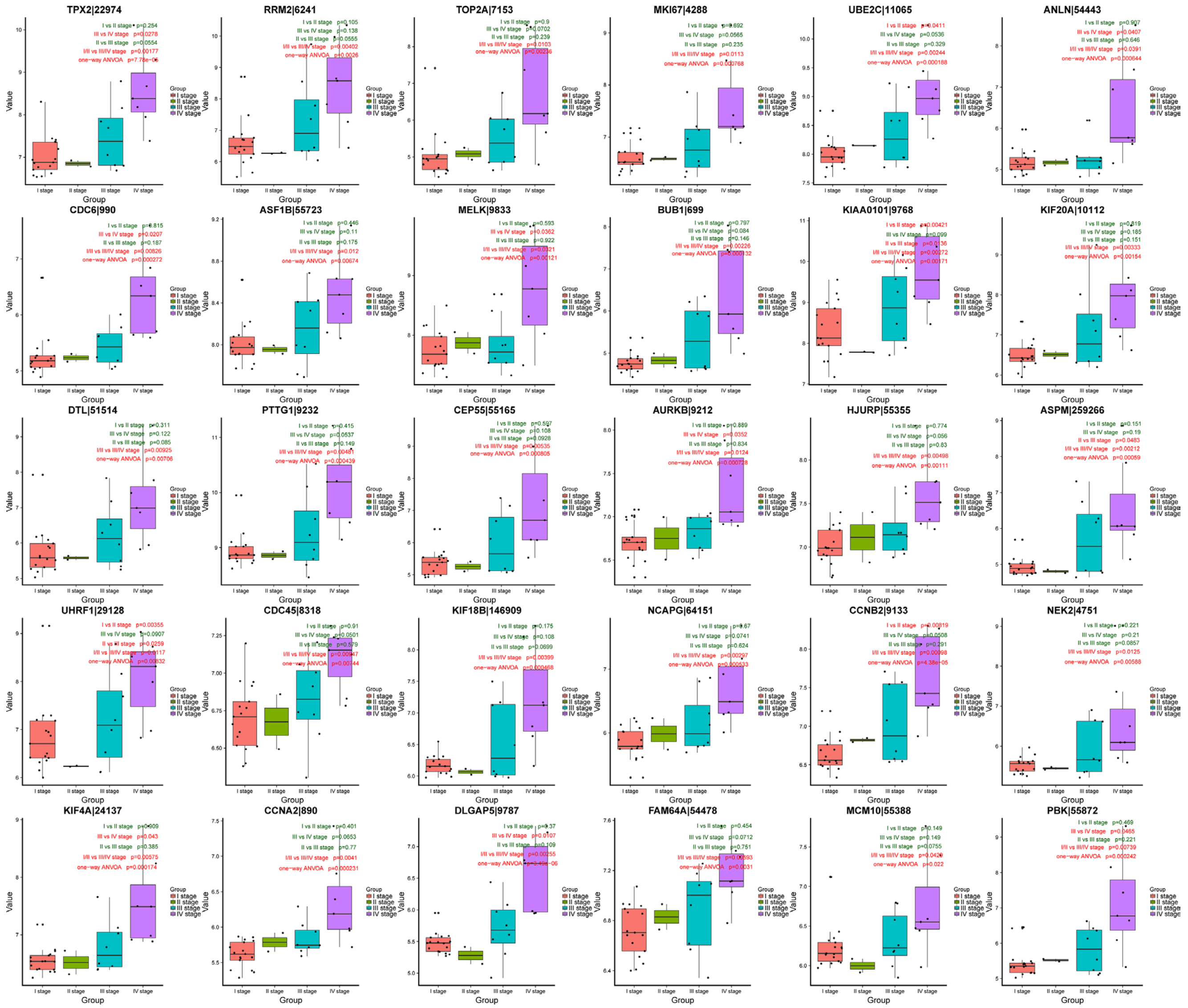

Supplementary Figure 4: Boxplots of 30 hub genes across different pathological stages in the validation set. The boxplots show the medians and dispersions of the samples of different pathological stages for each hub gene, where the scattered black spots represent the expression level of the genes. *P*-values are the results of independent sample *T*-test between pathological stage I and II, pathological stage III and IV, pathological stage II and III, pathological stage I/II and III/IV and one-way ANOVA for different pathological stages.

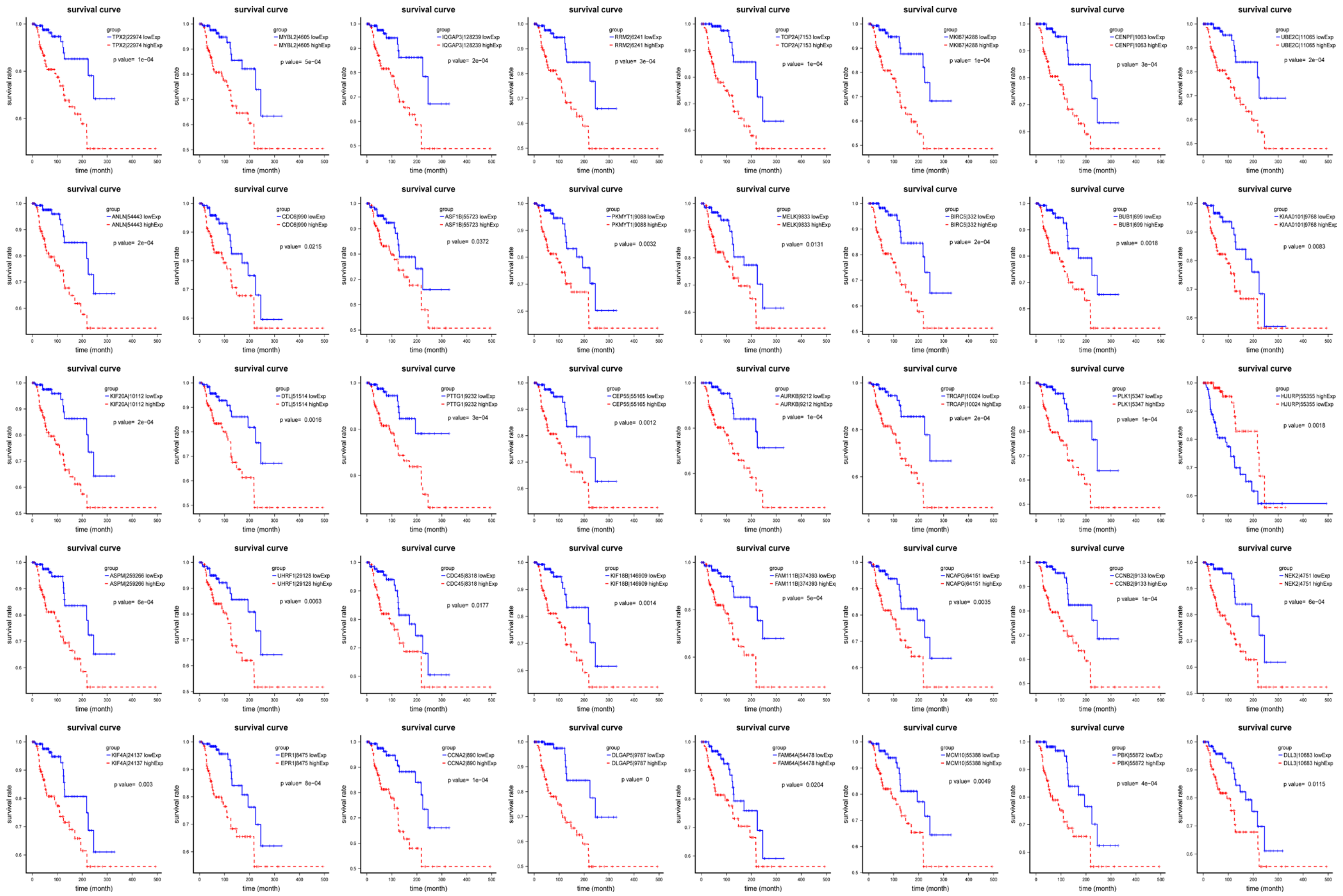

Supplementary Figure 5: Survival analyses on 40 hub genes in the TCGA data set. Survival curves for patients in different groups, solid blue lines represent high expression of hub genes and dashed red lines represent low expression of hub genes.

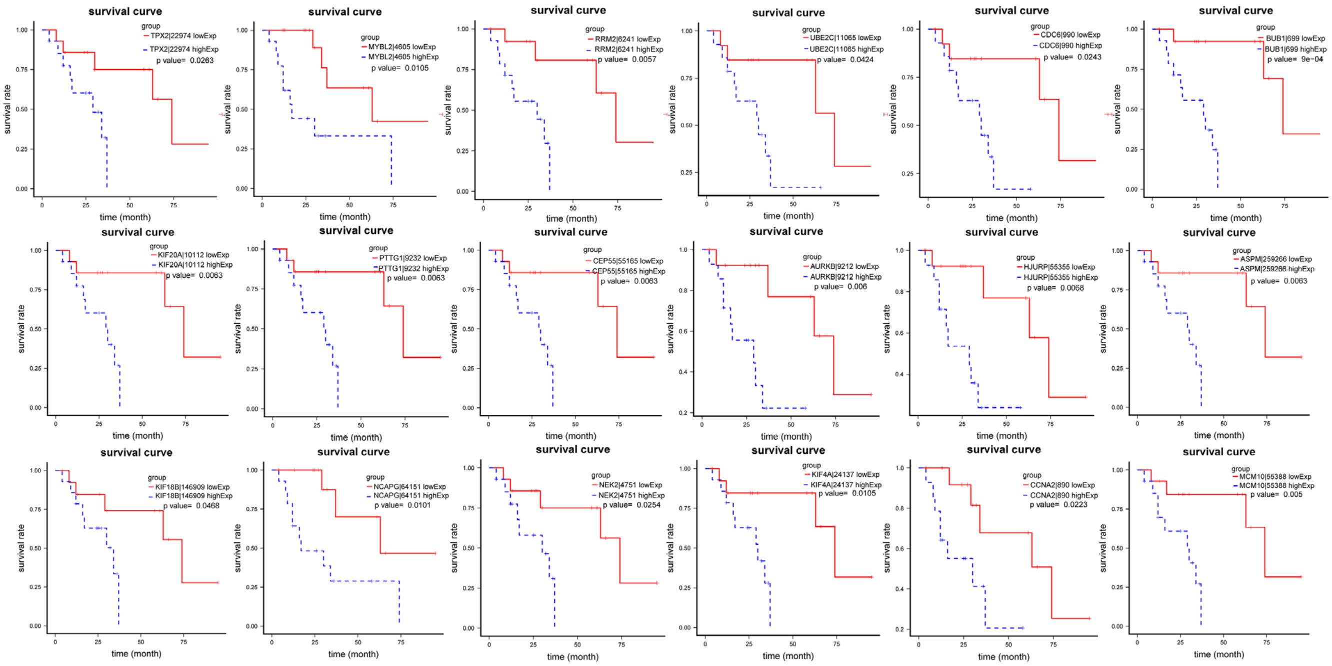

Supplementary Figure 6: Survival analyses on 18 hub genes in the validation set. Survival curves for patients in different groups, solid red lines represent high expression of hub genes and dashed blue lines represent low expression of hub genes.

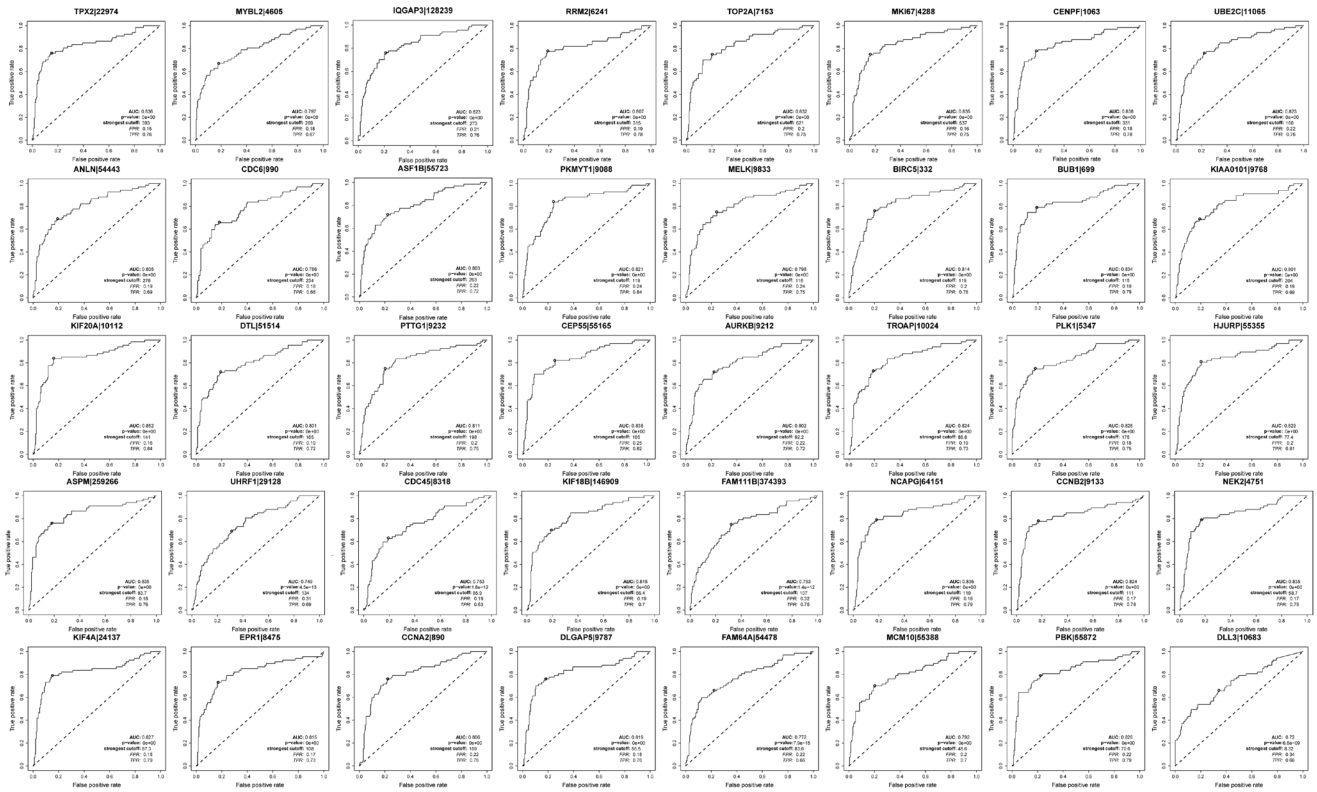

Supplementary Figure 7: ROC analyses on 40 hub genes in the TCGA data set. Receiver operating characteristic (ROC) curves and area under the curve (AUC) statistics to evaluate the diagnostic efficiency of the hub genes in TCGA to distinguish between localized and non-localized PRCC.

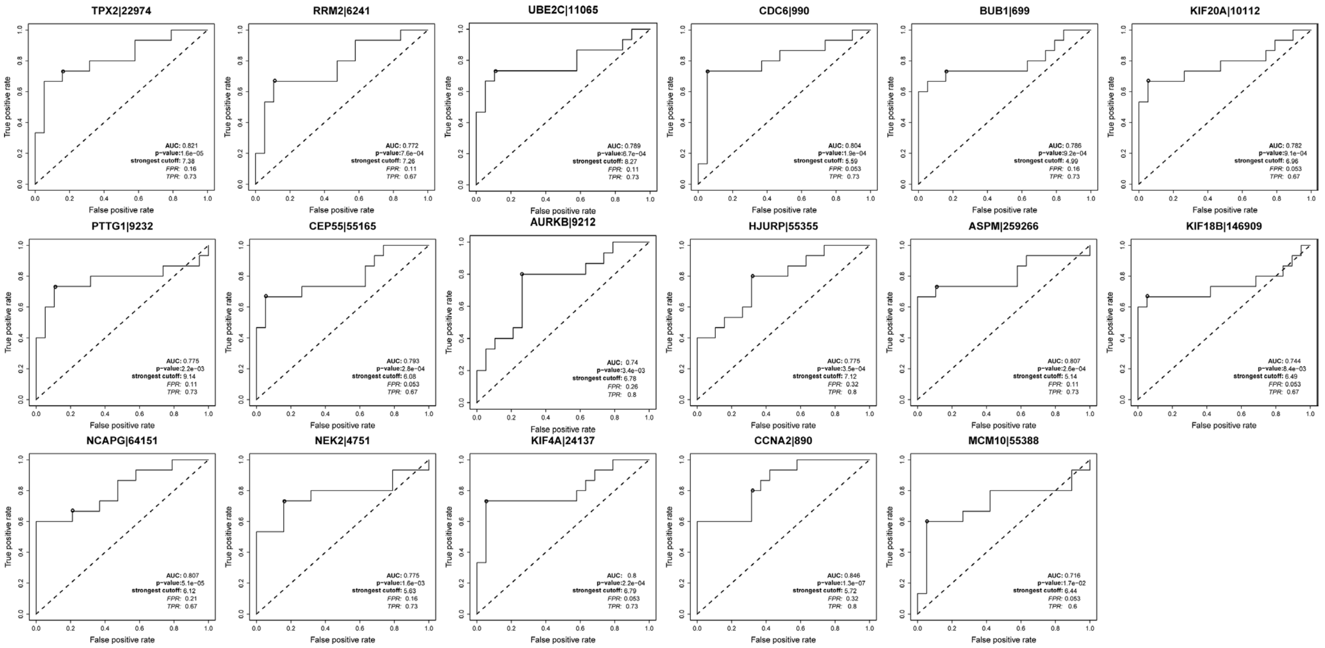

Supplementary Figure 8: ROC analyses on 17 hub genes in the validation set. Receiver operating characteristic (ROC) curves and area under the curve (AUC) statistics to evaluate the diagnostic efficiency of the hub genes in GSE2748 to distinguish between localized and non-localized PRCC.

Supplementary Table 1: Clinical information and numerically encoded results of PRCC patients in TCGA data set

Characteristics	Numerically encoded in R software	PRCC patients (*n* = 106)
Pathological stage		
Stage I	1	76
Stage II	2	7
Stage III	3	18
Stage IV	4	5
Pathology T stage		
pT1	1	78
pT2	2	8
pT3	3	19
pT4	4	1
Pathology N stage		
pN0	1	13
pN1	2	12
pN2	3	2
pNx	4	79
Pathology M stage		
pM0	1	37
pM1	2	3
pMx	3	66
Clinical stage		
Stage I	1	81
Stage II	2	7
Stage III	3	14
Stage IV	4	4
Clinical T stage		
cT1	1	82
cT2	2	10
cT3	3	13
cT4	4	1
Clinical N stage		
cN0	1	72
cN1	2	11
cN2	3	2
cNx	4	21
Clinical M stage		
cM0	1	89
cM1	2	2
cMx	3	15
Laterality		
bilateral	1	1
left	2	57
right	3	48
Tumor type		
Type I	1	47
Type II	2	59
Gender		
Male	1	76
Female	2	30
Age at diagnosis	28~85	28~85

Supplementary Table 2: Clinical features of PRCC patients in validation set GSE2748

Patient ID	Age (year)	Gender	Tumor-node-metastasis stage	Patient status	Survival (month)
P01	46	F	4	NA	NA
P02	59	M	1	NA	NA
P03	68	M	1	NA	NA
P04	32	M	1	NA	NA
P05	71	M	1	DOO	30
P06	70	M	3	NED	26
P07	72	F	1	DOO	8
P08	73	F	1	NED	25
P09	84	F	1	NED	27
P10	56	F	3	NED	13
P11	56	M	1	NED	13
P12	80	F	1	NED	4
P13	64	M	1	DOD	74
P14	44	M	3	NED	95
P15	76	M	1	NED	58
P16	72	M	3	DOD	17
P17	55	M	3	NED	30
P18	71	F	1	NED	32
P19	76	M	3	NED	36
P20	71	M	1	DOO	63
P21	80	F	2	NED	66
P22	53	M	1	NED	61
P23	54	M	4	DOD	12
P24	50	M	3	DOO	37
P25	44	M	4	AWC	12
P26	75	M	2	NA	NA
P27	74	M	1	NED	24
P28	37	M	1	DOO	12
P29	43	M	1	NA	NA
P30	63	M	3	DOO	34
P31	62	M	4	DOO	4
P32	56	M	4	DOD	16
P33	71	F	4	DOD	29
P34	49	F	4	DOD	9

*Last known status: DOD, died of disease; DOD, died of other causes; AWC, alive with cancer; NED, no evidence of disease.

Supplementary Table 3: Genes in the module are listed in gene-module. See Supplementary_Table_3.
