# Supplementary material for: Identifying biomarkers of papillary renal cell carcinoma associated with pathological stage by weighted gene co-expression network analysis
Source: Oncotarget. 2017 Mar 2;8(17):27904–14. doi: 10.18632/oncotarget.15842 (PMC5438617; doi:10.18632/oncotarget.15842)
Supplement: Supplementary file 2 [file oncotarget-08-27904-s002.docx]

| Supplementary Table 3: Genes in the module are listed in gene-module | |
| --- | --- |
| Gene Module | Gene ID |
| Black module | ENO2\|2026，C19orf33\|64073，CDKN2A\|1029，HS3ST2\|9956，MSC\|9242，HRK\|8739，RAB42\|115273，RGS20\|8601，SLC4A1\|6521，NPHS1\|4868，RALYL\|138046，NR4A3\|8013，SLC25A25\|114789，SUSD4\|55061，SLIT3\|6586，HSPB7\|27129，TRPV5\|56302，OLFM3\|118427，AOC3\|8639，NR4A1\|3164，ADAMTSL1\|92949，HCRTR2\|3062，SCTR\|6344，LMO3\|55885，LTBP1\|4052，COL14A1\|7373，LOC100302650\|100302650，TRPM6\|140803，APCDD1L\|164284，ANGPTL1\|9068，FMN2\|56776，ITGA8\|8516，SCUBE3\|222663，NEGR1\|257194，TSPAN2\|10100，NR4A2\|4929，PLN\|5350，MEIS2\|4212，LMOD1\|25802，PI16\|221476，PTPRQ\|374462，MYH11\|4629，SYNPO2\|171024，TBX2\|6909，MYOZ2\|51778，PTGS2\|5743，FAM26E\|254228，C1QTNF7\|114905，VWA5B1\|127731，GAS1\|2619，IQSEC3\|440073，ASB10\|136371，SGCZ\|137868，PDE3A\|5139，FOSB\|2354，CASQ2\|845，DUSP26\|78986，FGF10\|2255，TUSC5\|286753，FLRT2\|23768，WSCD2\|9671，MYOM1\|8736，FAM180A\|389558，FHL5\|9457，NKD1\|85407，PCDH7\|5099，VGLL3\|389136，LOC145820\|145820，SUSD5\|26032，ADRA2B\|151，OGN\|4969，ITGA9\|3680，MYOCD\|93649，CSF3\|1440，LDB3\|11155，CCL11\|6356，RXFP1\|59350，GDNF\|2668，DPP6\|1804，PID1\|55022，PDZRN4\|29951，UNC45B\|146862，MGAT4C\|25834，TRPV6\|55503，LOC284551\|284551，RSPO3\|84870，NTF3\|4908，FOXC2\|2303，DES\|1674 |
| Blue module | ASPHD1\|253982，BHLHE41\|79365，MUC12\|10071，TNFSF9\|8744，TIMP1\|7076，SCARB1\|949，CLDN1\|9076，TMSB10\|9168，GPRIN1\|114787，ANXA13\|312，PAQR4\|124222，RIN1\|9610，TPX2\|22974，MYBL2\|4605，IGFN1\|91156，ATHL1\|80162，KCNV1\|27012，IQGAP3\|128239，RRM2\|6241，TOP2A\|7153，MKI67\|4288，APBA2\|321，CENPF\|1063，UBE2C\|11065，CARD11\|84433，CDT1\|81620，ANLN\|54443，E2F1\|1869，CDC6\|990，ASF1B\|55723，NKX2-4\|644524，PKMYT1\|9088，MELK\|9833，BIRC5\|332，BUB1\|699，KIAA0101\|9768，KIF20A\|10112，GINS2\|51659，MNX1\|3110，BEAN\|146227，DTL\|51514，PTTG1\|9232，CEP55\|55165，TM4SF19\|116211，PRR7\|80758，AURKB\|9212，EZH2\|2146，TROAP\|10024，PLK1\|5347，HJURP\|55355，GOLGA7B\|401647，ASPM\|259266，UHRF1\|29128，CDC45\|8318，CLVS1\|157807，C16orf59\|80178，CDCA7\|83879，MLF1IP\|79682，PAQR6\|79957，KIF18B\|146909，FAM111B\|374393，NCAPG\|64151，TRPA1\|8989，CCNB2\|9133，NEIL3\|55247，PAQR9\|344838，NEK2\|4751，KIF4A\|24137，EPR1\|8475，CCNA2\|890，DLGAP5\|9787，FAM64A\|54478，TNNT1\|7138，MCM10\|55388，PKD1L2\|114780，SFN\|2810，FCHO1\|23149，BTBD16\|118663，ATP2A1\|487，PBK\|55872，DLL3\|10683，HRG\|3273，DUSP9\|1852，NPHS2\|7827，DDN\|23109，KNG1\|3827，SOST\|50964，AFM\|173，CRYAA\|1409，CLDN16\|10686，GPC3\|2719，SLC34A3\|142680，HS6ST2\|90161，BSND\|7809，TMEM207\|131920，ATP6V1C2\|245973，HMX2\|3167，FABP1\|2168，GPC5\|2262，UNCX\|340260，TYRO3\|7301，NAT8L\|339983，SEMG2\|6407，MRGPRF\|116535，KLK7\|5650，STRA6\|64220，SYT7\|9066，ODZ2\|57451，CAMK2A\|815，CA8\|767，HELT\|391723，ARC\|23237，WNT8B\|7479，LOC84856\|84856，RGS6\|9628，EPHB3\|2049，HPSE2\|60495，CACNA1S\|779，ZNF488\|118738，TUBAL3\|79861，CGA\|1081，KCNK13\|56659，SLC14A2\|8170，NXPH2\|11249，GRAMD1B\|57476，GAD1\|2571，DMRT2\|10655，GPBAR1\|151306，HMX3\|340784，SERPINA4\|5267，CLDN11\|5010，LOC647309\|647309，KIRREL2\|84063，BMP5\|653，FAM83B\|222584，PLXNA4\|91584，NRN1\|51299，DDX25\|29118，NMUR2\|56923，SH3GL3\|6457，FOXI1\|2299，WT1\|7490，SLC7A10\|56301，WIT1\|51352，GFRA3\|2676，GATA5\|140628，CDH10\|1008，CELF3\|11189，C19orf26\|255057，TMEM105\|284186，SOX10\|6663，ITLN2\|142683，LYPD6B\|130576，NR0B2\|8431 |
| Brown module | LPPR5\|163404，SPHK1\|8877，F12\|2161，SCG5\|6447，TRIM9\|114088，AKR1C2\|1646，AKR1C1\|1645，CHI3L2\|1117，LOC100131551\|100131551，SLC7A11\|23657，GCKR\|2646，LRIT2\|340745，SAA2\|6289，XKR3\|150165，WNK4\|65266，NELL1\|4745，EGF\|1950，GP2\|2813，GGT6\|124975，A2LD1\|87769，ALDOB\|229，ALB\|213，TMEM174\|134288，AGXT\|189，PLG\|5340，XPNPEP2\|7512，ASB15\|142685，AGMAT\|79814，ASS1\|445，CA4\|762，CLDN10\|9071，SORD\|6652，PDZD2\|23037，ASPDH\|554235，CYP4A11\|1579，HPD\|3242，MME\|4311，ACSF2\|80221，CYP8B1\|1582，ALDH6A1\|4329，AGPAT9\|84803，SUSD2\|56241，TCL6\|27004，TNNT2\|7139，ENPP6\|133121，BHMT\|635，CYP4F2\|8529，UPP2\|151531，DMGDH\|29958，PRODH2\|58510，MTTP\|4547，PIPOX\|51268，ADH1C\|126，RAP1GAP\|5909，FOLH1B\|219595，SLC6A19\|340024，DAO\|1610，SLC5A12\|159963，G6PC\|2538，ADH6\|130，IYD\|389434，HECW1\|23072，KMO\|8564，FRMD7\|90167，SLC13A3\|64849，APOM\|55937 KHK\|3795，C9orf66\|157983，CPNE6\|9362，DPYS\|1807，MIOX\|55586，DDC\|1644，C8orf80\|389643，SLC28A2\|9153，PCK1\|5105，CYP27B1\|1594，TTTY9B\|425057，AGXT2\|64902，SLC22A13\|9390，CUBN\|8029，DIO1\|1733，PCDH9\|5101，C2orf54\|79919，TREH\|11181，DNMT3L\|29947，HAO2\|51179，RDH12\|145226，SORCS2\|57537，KCNK10\|54207，SLC13A2\|9058，ALPL\|249，FRMD1\|79981，GPM6A\|2823，C1orf168\|199920，IL13RA2\|3598，TTC36\|143941，C19orf69\|100170765，DLGAP2\|9228，LOC100130691\|100130691，MOGAT2\|80168，OVCH2\|341277，SLC22A12\|116085，NR1I3\|9970，CYP4A22\|284541，GLTPD2\|388323，ATP6V1G3\|127124，UGT2A1\|10941，A1CF\|29974，SLC5A11\|115584，CPA1\|1357，PCDH15\|65217，CPN2\|1370，ZNF804B\|219578，TSPAN8\|7103，CYP4F3\|4051，ASPG\|374569，MGAM\|8972，UMODL1\|89766，MYCN\|4613，GABRB2\|2561，LOC148709\|148709，TPPP2\|122664，BPI\|671，SLITRK3\|22865，SLC30A8\|169026，FER1L6\|654463，ADH4\|127，LOC255167\|255167，DGKB\|1607，TTPA\|7274，UPF0639\|400224，SLC38A3\|10991，TMEM84\|283673，ART1\|417，MYOZ1\|58529，GYPA\|2993，TMIGD1\|388364，SLC6A4\|6532，KRT85\|3891，LPA\|4018，TMSB4Y\|9087，FGF5\|2250，KCNG2\|26251，CNTFR\|1271，RPRM\|56475，C14orf68\|283600，SCARA5\|286133，CAPZA3\|93661 |
| Green module | AHNAK2\|113146，HK2\|3099，TUBB3\|10381，C6orf174\|387104，SEZ6L2\|26470，TPK1\|27010，CREB5\|9586，LRRN4\|164312，TNFAIP6\|7130，C1orf186\|440712，APBB1IP\|54518，FHL1\|2273，PLCD3\|113026，CCND2\|894，PXDN\|7837，TMEM163\|81615，DNM1\|1759，SLFN13\|146857，SLC6A20\|54716，DDB2\|1643，ANXA1\|301，SYT12\|91683，PNMA2\|10687，HAVCR1\|26762，VSTM2L\|128434，SCEL\|8796，VCAN\|1462，STMN3\|50861，FAM189A1\|23359，SERPINE2\|5270，MET\|4233，FDXR\|2232，TNFRSF12A\|51330，ADCY2\|108，AMACR\|23600，HRH1\|3269，TFPI2\|7980，BBC3\|27113，SLC34A2\|10568，ELFN2\|114794，GRM5\|2915，FBLN7\|129804，MMP11\|4320，KCNT2\|343450，LRRC20\|55222，GALNT5\|11227，TBX15\|6913，MC1R\|4157，C6orf138\|442213，BRSK1\|84446，IL12RB2\|3595，SRCIN1\|80725，ONECUT2\|9480，CCDC135\|84229，PLEKHN1\|84069，EREG\|2069，LAMA3\|3909，SNAP25\|6616，EDA2R\|60401，MGC45800\|90768，ANKRD13B\|124930，CNR1\|1268，FANCA\|2175，ACSM1\|116285，WFDC5\|149708，WFDC12\|128488，RIMS2\|9699，BCO2\|83875，TRPM8\|79054，C3orf67\|200844，CDSN\|1041，VSTM2A\|222008，CELSR3\|1951，KCNH8\|131096，LRRC46\|90506，ANO4\|121601，GDAP1L1\|78997，HPCA\|3208，NCRNA00173\|100287569，FLJ32063\|150538，SLC12A1\|6557，CASR\|846，CLCNKB\|1188，EFHD1\|80303，CYFIP2\|26999，DNASE1\|1773，MFSD4\|148808，ACADSB\|36，IRX1\|79192，CLCNKA\|1187，SPTBN2\|6712，SIM2\|6493，CLDN8\|9073，HS6ST1\|9394，TMPRSS2\|7113，MPPED2\|744，CHRNA4\|1137，VSIG8\|391123，SLC8A1\|6546，SLC14A1\|6563，GRHL2\|79977，C14orf37\|145407，IL1RL1\|9173，SPOCK2\|9806，THSD7A\|221981，C1orf64\|149563，C2orf40\|84417，IGFBP2\|3485，C1orf226\|400793，SIM1\|6492，PSKH2\|85481，CA10\|56934，SLC4A8\|9498，GJD2\|57369，CLDN14\|23562，AGTR1\|185，RAG2\|5897，FRMD3\|257019，RIMBP2\|23504，FAM190A\|401145，RPS6KA6\|27330，C18orf34\|374864，NDST3\|9348，ENOX1\|55068，MARCH10\|162333，WEE2\|494551，C9orf70\|84850，TRPC6\|7225，GRIP2\|80852，PCSK9\|255738 |
| Greenyellow module | C1orf38\|9473，RASGEF1C\|255426，DNAH2\|146754，PIP\|5304，SFRP1\|6422，ACPP\|55，NFASC\|23114，TCF21\|6943，DCN\|1634，C7\|730，EHD3\|30845，RANBP3L\|202151，CNTN1\|1272，PTGER1\|5731，ADAMTSL2\|9719，PDGFRA\|5156，NR2F1\|7025，SVEP1\|79987，AVPR1A\|552，S1PR3\|1903，SIAH3\|283514，CRB2\|286204，EHF\|26298，TNNI1\|7135，GCGR\|2642，EGR3\|1960，ANGPT1\|284，GNA14\|9630，ADAMTS19\|171019，MFI2\|4241，LRRC55\|219527，PGR\|5241，PCDH18\|54510，IGSF10\|285313，PRSS22\|64063，HTR3B\|9177，GREM2\|64388，FGF7\|2252，DDR2\|4921，IL19\|29949，KIAA1210\|57481，SNCAIP\|9627，RUNX1T1\|862，PKNOX2\|63876，GRIK2\|2898，C15orf56\|644809 |
| Magenta module | ABCC3\|8714，RHBDF2\|79651，EEF1A2\|1917，LOC100126784\|100126784，UNC5A\|90249，PTPRH\|5794，CALB1\|793，SCNN1B\|6338，SLC9A4\|389015，AFAP1L2\|84632，ADH1B\|125，MECOM\|2122，TMEM45B\|120224，PTGER3\|5733，SLC26A7\|115111，MUC6\|4588，SLC43A1\|8501，NHLRC4\|283948，DACH1\|1602，LMX1B\|4010，B4GALNT3\|283358，KLHL3\|26249，SLC16A5\|9121，PCDH1\|5097，RBP2\|5948，COL4A6\|1288，EPB41L4B\|54566，SLC9A2\|6549，LDLR\|3949，TMEM61\|199964，PDE1A\|5136，SCNN1G\|6340，FAM69B\|138311，EPN3\|55040，PDK4\|5166，NIPAL1\|152519，IGDCC3\|9543，KIAA2022\|340533，BMPR1B\|658，ADCY1\|107，GATA3\|2625，FLJ45983\|399717，VAT1L\|57687，AVPR2\|554，SLC7A14\|57709，UNC5C\|8633，ODAM\|54959，RHBG\|57127，NOS1AP\|9722，NTRK1\|4914，DEFB132\|400830，ADH1A\|124，LOC286002\|286002，LMO1\|4004，HYAL4\|23553，DOC2B\|8447，IL11\|3589，LRRTM2\|26045 |
| Pink module | CNTN6\|27255，COL23A1\|91522，IGFBP6\|3489，PHLDA3\|23612，TNFSF12-TNFSF13\|407977，HSF4\|3299，TNNI3\|7137，HGFAC\|3083，CPNE7\|27132，SYCE1L\|100130958，WBSCR27\|155368，CD300A\|11314，C4orf48\|401115，SH2D5\|400745，KCNJ4\|3761，WBSCR26\|171022，DGCR9\|25787，LBX2\|85474，LOC100128076\|100128076，MESP2\|145873，LOC284578\|284578，EMCN\|51705，MUC15\|143662，TIMP3\|7078，CRHBP\|1393，SEMA3G\|56920，TEK\|7010，ATP12A\|479，TFAP2B\|7021，IGFBP5\|3488，SLC9A3\|6550，TMEM213\|155006，PODXL\|5420，NES\|10763，PTPRB\|5787，C7orf41\|222166，PRDM16\|63976，RASL11B\|65997，CLIC5\|53405，LOC25845\|25845，CLEC3B\|7123，ATP6V1B1\|525，FLJ42875\|440556，C16orf11\|146325，NUAK2\|81788，TSPAN7\|7102，CD34\|947，HSPA2\|3306，HOXD8\|3234，TMTC1\|83857，C6orf176\|90632，RAMP3\|10268，SEMA6D\|80031，PDE2A\|5138，GJA5\|2702，MYO3B\|140469，MMRN2\|79812，ERBB4\|2066，TMEM178\|130733，ROBO4\|54538，PPP1R16B\|26051，HSD11B2\|3291，TIE1\|7075，ATP1B2\|482，WNT9B\|7484，DPEP1\|1800，C4orf31\|79625，SLC12A3\|6559，CEACAM1\|634，KIF26A\|26153，NR3C2\|4306，CALCA\|796，ST6GALNAC3\|256435，STC1\|6781，PIK3C2G\|5288，EYA4\|2070，SLC16A2\|6567，GADL1\|339896，KDR\|3791，GATA2\|2624，LGI2\|55203，FLT4\|2324，RNF150\|57484，RERGL\|79785，DAAM2\|23500，LRRC2\|79442，TMEM30B\|161291，TMEM204\|79652，GABRA2\|2555，LDB2\|9079，LNX1\|84708，MST1P9\|11223，ARHGEF15\|22899，GPR116\|221395，EXOC3L2\|90332，KCNJ1\|3758，LRRN2\|10446，FOLH1\|2346，LOC145837\|145837，PAK6\|56924，NOS3\|4846，CYYR1\|116159，REN\|5972，PCDH12\|51294，ERG\|2078，SELE\|6401，GSTM3\|2947，APLNR\|187，FCN3\|8547，CRABP1\|1381，CCL14\|6358，ROS1\|6098，NTNG1\|22854，ABCA4\|24，TYRP1\|7306，UBE2QL1\|134111，C16orf89\|146556，GPIHBP1\|338328，GALNTL2\|117248，NTS\|4922，HSPA12B\|116835，SCUBE1\|80274，SELP\|6403，ATP6V0A4\|50617，CACNA1H\|8912，AAA1\|404744，GABRA4\|2557，HOXD3\|3232，ATP1A2\|477，FAM3B\|54097，SALL3\|27164，SCN7A\|6332，ADCY4\|196883，SOX18\|54345，MYCT1\|80177，HOXD10\|3236，FGF9\|2254，MFSD6L\|162387，NRIP2\|83714，OPCML\|4978，LOC389493\|389493，C4orf32\|132720，BCL6B\|255877，LYVE1\|10894，FXYD4\|53828，CYP4X1\|260293，AGTR2\|186，RAPGEF4\|11069，CNGA1\|1259，FOLR3\|2352，EMID2\|136227，SYP\|6855，RAET1E\|135250，C2orf72\|257407，FLJ42709\|441094，RASGRF2\|5924，SLC45A1\|50651，USHBP1\|83878，FAM184A\|79632，C20orf160\|140706，JAM2\|58494，PDE8B\|8622，SOX7\|83595，BDKRB2\|624，C8orf12\|83656，C12orf59\|120939，TBXA2R\|6915，SYN3\|8224，TCEAL2\|140597，LRRC10B\|390205，KCNN3\|3782，UNC13C\|440279，LOC441177\|441177，KANK4\|163782，PLA2G4F\|255189，KCNN2\|3781，RGS7BP\|401190，ZNF366\|167465，SGIP1\|84251，KIF5C\|3800，SVOPL\|136306，KCNJ9\|3765，C11orf16\|56673，LHX1\|3975，HRC\|3270，PRMT8\|56341，SOX17\|64321，FXYD1\|5348，C3orf32\|51066，RASSF9\|9182，GRRP1\|79927，CHRM3\|1131，FABP4\|2167，BDKRB1\|623，HSN2\|378465，PYGM\|5837，PLAT\|5327，COL25A1\|84570，LYPD6\|130574，GABRP\|2568，LRRC70\|100130733，ADAMTS6\|11174，DRD1\|1812，GPR77\|27202，FAM162B\|221303，VGLL1\|51442 |
| Purple module | ABCA12\|26154，PRAME\|23532，MDK\|4192，CORO6\|84940，DOC2A\|8448，ATP8B3\|148229，IGDCC4\|57722，C20orf46\|55321，DNAJB13\|374407，LOC284749\|284749，GPR172B\|55065，SLC17A2\|10246，PRKCG\|5582，CATSPER1\|117144，F11\|2160，ESRRB\|2103，CLDN19\|149461，ESRRG\|2104，CYP2B6\|1555，SLC4A9\|83697，SLC26A4\|5172，HPCAL4\|51440，THY1\|7070，GPR98\|84059，MCCD1\|401250，SLC2A12\|154091，MYLK3\|91807，GRM1\|2911，HMGCS2\|3158，CHP2\|63928，APOH\|350，CES3\|23491，HAO1\|54363，FAM184B\|27146，C20orf54\|113278，PNPLA1\|285848，TNNC1\|7134，TBL1Y\|90665，LGSN\|51557，DACH2\|117154，SYN2\|6854，C5orf27\|202299，ADIPOQ\|9370，ACSBG2\|81616，C6orf146\|222826，LOC285780\|285780，C1orf125\|126859，KRTAP5-8\|57830，CLUL1\|27098，MYOC\|4653，CDC20B\|166979，SLC13A5\|284111 |
| Red module | SPON2\|10417，HSPB8\|26353，TUBB4\|10382，C2orf89\|129293，CDH17\|1015，CLGN\|1047，SYT14\|255928，AKR1D1\|6718，TMEM72\|643236，PLCL1\|5334，CGNL1\|84952，FBP1\|2203，PLA2R1\|22925，TFCP2L1\|29842，SFXN2\|118980，SLC5A3\|6526，SLC22A8\|9376，LOC643008\|643008，SELENBP1\|8991，SLC36A2\|153201，FAM151A\|338094，MRO\|83876，GPX3\|2878 SLC7A8\|23428，NRK\|203447，ANK2\|287，RNF152\|220441，PARM1\|25849，GPR155\|151556，PROZ\|8858，FREM1\|158326，ARG2\|384，MAN1C1\|57134，LPPR1\|54886，PROX1\|5629，DPT\|1805，SLC7A9\|11136，MAPK4\|5596，FAM107A\|11170，C12orf34\|84915，PRLR\|5618，SERPINA5\|5104，CTXN3\|613212，SOCS2\|8835，OXGR1\|27199，ELF5\|2001，LOC340094\|340094，SLC5A2\|6524，PM20D1\|148811，PAH\|5053，MRVI1\|10335，NTRK2\|4915，HPGD\|3248，PEG3\|5178，ST6GAL1\|6480，NT5C1A\|84618，RNF186\|54546，SSTR2\|6752，GPR113\|165082，FAM46B\|115572，NCRNA00175\|378832，GPR182\|11318，ARSF\|416，ACSL6\|23305，CHAC1\|79094，PNPLA3\|80339，KCNJ3\|3760，ACOT12\|134526，PRSS35\|167681，UGT1A9\|54600，CCBE1\|147372，ZNF521\|25925，TMC1\|117531，C17orf88\|23591，SLC22A6\|9356，ZP2\|7783，PPP2R2B\|5521，FSTL4\|23105，C22orf45\|646023，AMPH\|273，PGAM2\|5224，DNM3\|26052，CYP3A4\|1576，PPAPDC1A\|196051，INPP5J\|27124，LRRC52\|440699，KCNA1\|3736，WNK3\|65267，CKM\|1158，MMRN1\|22915，STON1-GTF2A1L\|286749，ISX\|91464，VIT\|5212 |
| Turquoise module | ITGAX\|3687，TREM2\|54209，ALOX5\|240，SDS\|10993，APOC1\|341，TYMP\|1890，DGCR5\|26220，CD68\|968，IFI27\|3429，LILRB4\|11006，CD70\|970，ADORA3\|140，PLA2G7\|7941，CCL18\|6362，SIGLEC8\|27181，KDELC1\|79070，IL32\|9235，NNMT\|4837，HHLA2\|11148，CHIT1\|1118，LAIR1\|3903，ODF3B\|440836，FCER1G\|2207，CSF3R\|1441，CHRNA1\|1134，LAPTM5\|7805，ALOX15B\|247，FCGR3A\|2214，CCDC88B\|283234，TYROBP\|7305，MYO1F\|4542，RGS1\|5996，SLC37A2\|219855，PARVG\|64098，TRPM2\|7226，LY86\|9450，HAMP\|57817，LCN2\|3934，MARCO\|8685，CXCL6\|6372，SLC1A3\|6507，C3\|718，HSPA7\|3311，APOB48R\|55911，LSP1\|4046，PIK3R5\|23533，SPAG4\|6676，CRYAB\|1410，OSCAR\|126014，PLA2G2D\|26279，FCGR2B\|2213，HTRA4\|203100，C1QB\|713，ITGAD\|3681，HLA-J\|3137，FABP6\|2172，GAL3ST4\|79690，PKD2L1\|9033，NCF1\|653361，OTOA\|146183，CXCL5\|6374，C15orf48\|84419，CCDC109B\|55013，LILRA2\|11027，CD300LF\|146722，CA9\|768，PRAM1\|84106，ADAMTS14\|140766，DNAJC5B\|85479，FCGR1A\|2209，IGSF6\|10261，DHRS9\|10170，BCL2A1\|597，BTK\|695，LPAR5\|57121，ARHGAP22\|58504，DPEP2\|64174，KRT86\|3892，SULT4A1\|25830，STAC3\|246329，SIGLEC10\|89790，FCGR1B\|2210，CLEC5A\|23601，LILRB1\|10859，ADAMDEC1\|27299，DNASE2B\|58511，BIRC7\|79444，PVT1\|5820，LILRA6\|79168，HK3\|3101，LILRB3\|11025，TEX11\|56159，FCGR2C\|9103，CD180\|4064，LOC653786\|653786，LILRA4\|23547，SIGLEC7\|27036，CSTA\|1475，PIK3R6\|146850，SIGLEC9\|27180，TM7SF4\|81501，NCF1C\|654817，KCTD4\|386618，UMOD\|7369，KCNJ10\|3766，AQP2\|359，INSRR\|3645，FGF1\|2246，SLC34A1\|6569，IRX2\|153572，UPB1\|51733，AQP3\|360，LPL\|4023，GSTA2\|2939，PCOLCE2\|26577，KCNE1\|3753，DNASE1L3\|1776，CXCL12\|6387，FLRT1\|23769，GSTA1\|2938，C2orf71\|388939，GHR\|2690，KCNQ1\|3784，GJA3\|2700，FAM169A\|26049，CR1\|1378，SLC23A3\|151295，RASD1\|51655，ANGPTL3\|27329，CCNI2\|645121，C6orf105\|84830，ITLN1\|55600，CDA\|978，ST8SIA6\|338596，OSTalpha\|200931，ZYG11A\|440590，CDH3\|1001，MYF6\|4618，APOC3\|345，PLA2G12B\|84647，JPH4\|84502，PLCXD3\|345557，PROM2\|150696，BNIPL\|149428，C14orf50\|145376，PLA2G3\|50487，LOC340508\|340508，ATP6V0D2\|245972，CPAMD8\|27151，OLIG1\|116448，OIT3\|170392，C11orf53\|341032，ADRB1\|153，B3GALT2\|8707，C10orf55\|414236，MT1H\|4496，CAPN8\|388743 |
| Grey module | HAGHL\|84264，CCDC78\|124093，PSORS1C1\|170679，CDHR1\|92211，TBC1D3G\|654341，CST9\|128822，TDGF1\|6997，SLC7A13\|157724，TACR3\|6870，TCL1B\|9623，TDGF3\|6998，CYP1A1\|1543，C9orf84\|158401，FAM5C\|339479，CSMD3\|114788 |
